# Protocol for HIV-1 budding control by inducible inhibition of ESCRT-III

**DOI:** 10.1016/j.xpro.2025.103808

**Published:** 2025-05-13

**Authors:** Haiyan Wang, Winfried Weissenhorn, Cécile Boscheron

**Affiliations:** 1 University Grenoble Alpes, CEA, CNRS, Institut de Biologie Structurale (IBS), Grenoble, France

**Keywords:** cell biology, microbiology, microscopy

## Abstract

We present a protocol for temporal inhibition of HIV-1 virus-like particle (VLP) release using ESCRT-III proteins fused to the Hepatitis C virus NS3 protease. These fusion proteins function like wild-type ESCRT-III but convert into dominant-negative inhibitors upon addition of the NS3 inhibitor Glecaprevir. The procedure involves co-transfection of Gag and CHMP-NS3-Green plasmids into HEK293 or HeLa cells, followed by drug treatment. Steps for protein expression analysis, VLP quantification by immunoblotting, and live-cell imaging of VLP release kinetics are included.

For complete details on the use and execution of this protocol, please refer to Wang et al.^1^

## Highlights

• Instructions for drug-inducible temporal control of HIV-1 VLP release in cell culture
• Guidelines for titrating CHMP-NS3 plasmid dosage to modulate ESCRT-III inhibition
• Procedures for quantifying HIV-1 VLP release using immunoblotting of cell supernatants
• Steps for high-resolution live imaging and kinetic analysis of single-cell VLP budding events

## Abstract (editor highlights)

Publisher’s note: Undertaking any experimental protocol requires adherence to local institutional guidelines for laboratory safety and ethics.

## Graphical abstract

**Figure:**
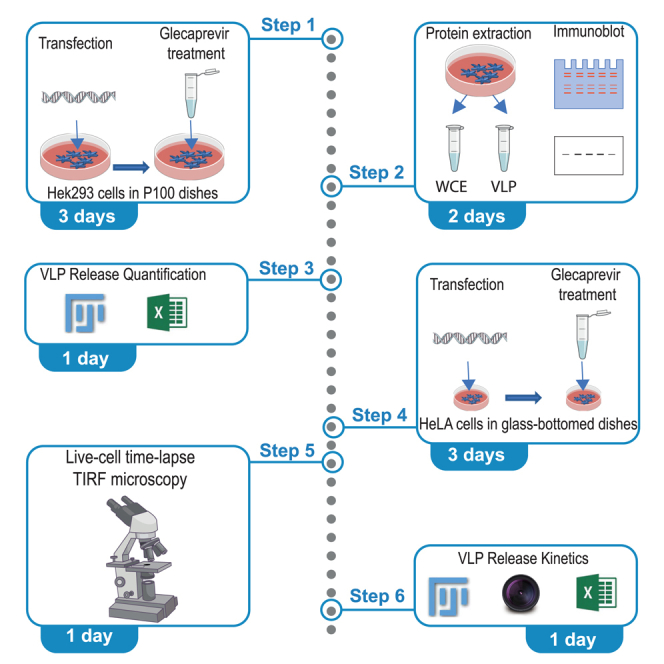

## Before you begin

The final stage of the retroviral life cycle, such as HIV-1, involves exiting host cells by budding from the plasma membrane thereby acquiring their membrane envelope.^2^ To achieve this, retroviruses recruit the cellular ESCRT (Endosomal Sorting Complexes Required for Transport) machinery, which catalyzes virus release via membrane fission.^3^^,^^4^^,^^5^^,^^6^ ESCRT-III and VPS4 form a core membrane remodeling complex that is highly conserved across archaea, bacteria, and eukaryotes.^3^^,^^7^^,^^8^^,^^9^ Although humans express twelve ESCRT-III proteins, only a small subset namely CHMP4, CHMP2B, and CHMP2A/CHMP3 is required for HIV-1 budding.^10^^,^^11^ ESCRT-III proteins shuttle between an inactive cytosolic closed conformation^12^^,^^13^ and an activated open conformation. The latter polymerizes on membranes into filaments with diverse architectures.^4^^,^^14^^,^^15^^,^^16^^,^^17^^,^^18^^,^^19^^,^^20^^,^^21^^,^^22^^,^^23^ Consistent with ESCRT-III remodeling *in vivo* by VPS4,^24^^,^^25^ ESCRT-III CHMP2A-CHMP3 polymers are remodeled and constricted *in vitro* by VPS4B^26^^,^^27^ up to the point of membrane cleavage.^16^ Expression of HIV-1 Gag is sufficient to drive assembly and budding of virus-like particles from the plasma membrane,^28^^,^^29^ recruiting ESCRT-III for release.^30^ Live-cell imaging has revealed the sequence of ESCRT-III recruitment during HIV-1 budding,^31^^,^^32^^,^^33^^,^^34^ but detailed structural information on native ESCRT-III at budding sites remains elusive. This is due to challenges in capturing narrow (∼50 nm) bud necks and the transient, short-lived nature of ESCRT-III recruitment, which lasts only a few minutes.^33^

To prolong the residence time of CHMP2A, CHMP3, and CHMP4B and slow down their exchange and/or remodeling, we developed an assay that converts these proteins into VPS4-deficient variants. We employ CHMP2A, CHMP3, and CHMP4B proteins fused to the Hepatitis C NS3 protease, which behave like wild type due to auto-cleavage. However, in the presence of the protease inhibitor Glecaprevir, non-cleaved CHMP2A, CHMP3, and CHMP4B-NS3 are transformed into budding inhibitors.^1^ This assay offers precise control over the timing of non-cleaved CHMP2A-NS3, CHMP3-NS3 and CHMP4B-NS3 protein expression, enabling targeted inhibition of specific stages of HIV-1 budding. Additionally, the level of inhibition can be modulated by adjusting CHMP-NS3 plasmid dosage during transfection. The following protocol describes the step-by-step implementation of this assay, including transfection of HEK293 and HeLa cells with auto-cleavable CHMP2A, CHMP3, and CHMP4B fusion proteins linked to the hepatitis C virus NS3 protease. It also details the procedures for quantifying VLP release inhibition via immunoblotting and monitoring VLP release kinetics in individual cells through high-resolution video microscopy. Although initially designed to investigate HIV-1 budding in HEK293 and HeLa cells, this assay is versatile and can be adapted to explore a variety of ESCRT-III-driven cellular processes in different cell lines.

## Key resources table

**Table:** 

REAGENT or RESOURCE	SOURCE	IDENTIFIER
**Antibodies**		

Rabbit polyclonal anti-Flag antibody, diluted 1:1,000	Sigma-Aldrich	Cat#F7625
Mouse monoclonal anti-human immunodeficiency virus type 1 (HIV-1) p24 antibody, diluted 1:1,000	BEI Resources	Cat#ARP-3537
Rabbit polyclonal anti-GFP antibody, diluted 1:1,000	Invitrogen	Cat#A11122
Anti-mouse IgG HRP, diluted 1:5,000	Sigma-Aldrich	Cat#A9044
Anti-rabbit IgG HRP, diluted 1:5,000	Sigma-Aldrich	Cat#A0545

**Biological samples**		

Fetal bovine serum	Life Technologies	Cat#10270106

**Chemicals, peptides, and recombinant proteins**		

DPBS	Life Technologies	Cat#14190169
L-glutamine	Life Technologies	Cat#25030024
DMEM HG Pyr	Life Technologies	Cat#41966052
Trypsin-EDTA	Life Technologies	Cat#25200056
Poly-lysine	Sigma-Aldrich	Cat#P4832
Glecaprevir	CliniSciences	Cat#HY-17634-10mg
Protein ladder : page-ruler prestained	Life Technologies	Cat#26619
Ethanol 96%	CARLO ERBA	Cat#528151
Glycine	Euromedex	Cat#26-128-6405
SDS	Euromedex	Cat#BI-SB0485
Sucrose	Sigma-Aldrich	Cat#S0389
Tris base	Euromedex	Cat#200923-A
30% acrylamide	Euromedex	Cat#EU0088-B
TEMED	Euromedex	Cat#50406
PBS 10x	Euromedex	Cat#ET330A
Tween 20	Euromedex	Cat#2001-B
Skimmed milk	Régilait	Cat#Ecrémé
cOmplete EDTA-free	Roche	Cat#8141500
Ammonium persulfate	Euromedex	Cat#EU0009

**Critical commercial assays**		

NucleoBond Xtra Midi endotoxin free	MACHEREY-NAGEL	Cat#740420.10
jetPRIME transfectant	Ozyme	Cat#POL101000046
Clarity western ECL	Bio-Rad	Cat#1705060

**Deposited data**		

Tracking dataset for Gag-mCherry spots	https://entrepot.recherche.data.gouv.fr/dataset.xhtml?persistentId=doi:10.57745/69UNAM	https://entrepot.recherche.data.gouv.fr/dataset.xhtml?persistentId=doi:10.57745/69UNAM

**Experimental models: Cell lines**		

HeLa cells	ATCC	Cat#CCL-2
HEK-293T cells	ATCC	Cat#CRL-3216

**Recombinant DNA**		

pCHMP2A-NS3-Green	Wang et al.^1^	Addgene Cat#223440
pCHMP3-NS3-Green	Wang et al.^1^	Addgene Cat#223527
pCHMP4B-NS3-Green	Wang et al.^1^	Addgene Cat#223528
pCHMP2A-mut-NS3-Green	Wang et al.^1^	Addgene 223529
pCHMP3-mut-NS3-Green	Wang et al.^1^	Addgene Cat#223530
pCHMP4B-mut-NS3-Green	Wang et al.^1^	Addgene Cat#223531
pCHMP2A-NS3-Blue	Wang et al.^1^	Addgene Cat#223532
pCHMP3-NS3-Blue	Wang et al.^1^	Addgene Cat#223533
pCHMP4B-NS3-Blue	Wang et al.^1^	Addgene Cat#223534
Vpu-expressing plasmid	Nguyen, K. L., et al.^35^	BEI resources Cat#ARP-176
pGag-mCherry	Jouvenet et al.^28^	
pCG-GagRevInd-7ires-puro	Kotsopoulou et al.^36^	

**Software and algorithms**		

GraphPad Prism	GraphPad	https://www.graphpad.com/
Fiji	Schindelin et al.^37^	https://imagej.net/software/fiji/downloads
Icy	de Chaumont et al.^38^	https://icy.bioimageanalysis.org/

**Other**		

Micro dishes 35 mm	Biovalley	Cat#81158
P100 tissus culture dish	Corning	Cat#353003
Cell lifter	VWR	Cat#99010
Nitrocellulose blotting membrane	Amersham	Cat#10600008
Whatman 3MM CHR	Cytiva	Cat#3030-917
Semi-dry transfer tank	Bio-Rad	Cat#170-4155
ChemiDoc	Bio-Rad	
Electrophoresis chambers	Bio-Rad	Cat#1658004

## Materials and equipment

**Table:** Cell culture medium

Reagent	Final concentration	Amount
DMEM	N/A	44.5 mL
Fetal serum bovine	N/A	5 mL
L-Glutamine	N/A	0.5 mL
**Total**	**N/A**	**50 mL**

Store at −4°C, protected from light, for up to one week.

**Table:** Glecaprevir stock solution

Reagent	Final concentration	Amount
Glecaprevir	10 mM	10 mg
DMSO	N/A	1.2 mL
**Total**	**N/A**	**1.2 mL**

Store at −80°C for up to six months in aliquots. Avoid repeated freeze-thaw cycles.

**Table:** Lysis buffer

Reagent	Final concentration	Amount
Tris-HCl 1 M pH 7.5	20 mM	2 mL
NaCl 5 M	150 mM	3 mL
MgCl_2_ 1 M	1 mM	0.1 mL
Triton X-100	1%	1 mL
cOmplete EDTA-free pro-tease inhibitor	N/A	N/A
H_2_O		93.9 mL
**Total**		**100 mL**

Store at 20°C for up to six months. Before use, add one tablet of protease

Inhibitor to 50 mL of lysis buffer.

**Table:** 20% sucrose solution

Reagent	Final concentration	Amount
Sucrose	20% (w/v)	20 g
H_2_O		100 mL
**Total**		**100 mL**

Store at 4°C for up to one months.

**Table:** 10% SDS-Polyacrylamide resolving gel solution

Reagent	Final concentration	Amount
Tris-HCl 1.5 M pH 8.5	375 mM	2.5 mL
30% Acrylamide mix	10%	3.3 mL
10% SDS	0.1%	0.1 mL
10% ammonium persulfate	0.1%	0.1 mL
TEMED	0.04%	0.004 mL
H_2_O		4 mL
**Total**		**10 mL**

Prepared extemporaneously.

**Table:** 5% SDS-Polyacrylamide stacking gel solution

Reagent	Final concentration	Amount
Tris-HCl 1 M pH 6.5	mM	0.25 mL
30% Acrylamide mix	5%	0.33 mL
10% SDS	0.1%	0.02 mL
10% ammonium persulfate	0.1%	0.02 mL
TEMED	0.04%	0.002 mL
H_2_O		1.4 mL
**Total**		**2 mL**

Prepared extemporaneously.

**Table:** 10x Tris-Glycine-SDS running gel solution

Reagent	Final concentration	Amount
Tris-HCl base	250 mM	30.284 g
Glycine	1.92 M	144.13 g
SDS	1%	10 g
H_2_O		1 L
**Total**		**1 L**

To prepare 1 liter of 1X Tris-Glycine-SDS running buffer, dilute 100 mL of 10X stock with 900 mL of distilled water (dH_2_O). Store at 20°C for up to six months.

**Table:** Transfer gel solution

Reagent	Final concentration	Amount
10x Tris-Glycine-SDS	1x	100 mL
Ethanol	20%	200 mL
H_2_O		700 mL
**Total**		**1 L**

Prepared extemporaneously.

**Table:** PBST solution

Reagent	Final concentration	Amount
PBS 10x	1x	50 ml
Tween 20	0.1%	5 mL
H_2_O		445 mL
**Total**		**500 mL**

Prepared extemporaneously.

**Table:** PBST milk solution

Reagent	Final concentration	Amount
PBST	1x	50 ml
Skimmed milk	5%	2.5 g
**Total**		**50 mL**

Prepared extemporaneously.

**Table:** 4 fold loading buffer

Reagent	Final concentration	Amount
Tris base 1 M pH 6.8	50 mM	2.5 mL
Bromophenol blue	0.1%	200 mg
SDS 10%	2%	10 mL
Glycerol 50%	10%	10 mL
2-mercaptoethanol 99%	5%	
**Total**	**N/A**	**25 mL**

Store at 20°C for up to one year. Before use, add 50 μL of 2-mercaptoethanol to 950 μL of 4x loading buffer.

### TIRF video-microscopy setup

#### Microscope stand and motorized components

We used a fully automated inverted digital fluorescence microscope (iMIC 2.0, Till-Photonics).

Illumination control: The system does not include a conventional shutter; instead, it utilizes the Polytrope II module (Till-0000-585-20024, Till-Photonics), a galvanometric device enabling rapid switching between epifluorescence and laser sources.

Focusing system: Equipped with a voice coil focus drive and piezo-based nano drive (Till-Photonics).

Focusing mechanism: Single piezo drive controlling all objectives, providing a Z-range of 250 μm with a resolution of 50 nm (Till-Photonics).

Hardware-based focus maintenance: Integrated Focus Clamp (TIL S30-25043, Till-Photonics).

### Illumination

Light source: Diode lasers 405 nm (120 mW), 488 nm (100 mW), 561 nm (115 mW), 642 nm (140 mW).

Laser source manufacturer: SOLE-6, OMICRON.

TIRF Configuration: Multi-point TIRF SW Module (Til-550-25020) with Yanus IV scan head (TIL-585-20028) and Polytrope II galvanometric device (Till-0000-585-20024, Till-Photonics).

### Wavelength selection

Excitation filters: Quad-band excitation filter (Till-555-25019).

Center wavelength and bandwidth (FWHM): 405-488-561-640 nm.

Emission filters:
  For 488 nm laser: 525/50 nm bandpass (F47-525, Chroma).
  For 561 nm laser: 600/50 nm bandpass (F47-601, Chroma).

### Optics

Objective lens: Alpha-Plan-Apochromat. Magnification: 63x. Numerical aperture (NA): 1.46. Part number: 420780-9970-000. Manufacturer: Carl Zeiss.

Contrast-enhancing technique / Bright-field source: RGB LED source (Till-Photonics).

Condenser numerical aperture: NA 0.5.

### Detection

Camera: iXon U897 EMCCD (Andor)

Binning: 1.

Tube refractor: 2.55.

Bit depth & gain: 14-bit, pre-amp gain 1.

EM gain: 200.

Pixel size: 0.099 μm.

### Acquisition software

Software: Live Acquisition, version 2.7.0.16 (Till-Photonics). The system saves images in 16-bit TIFF format.

## Step-by-step method details

### Steps for co-transfecting Gag and CHMP-NS3-Green plasmids into HEK293 cells and applying protease inhibitor treatment in P100 dishes

**Timing: 3 days (steps 1 to 3)**

**CRITICAL:** Decontaminate the hood, bench surface, and pipettes with 70% ethanol prior to use to prevent any potential contamination.

***Note:*** The plasmid amounts described here have been optimized to achieve strong inhibition under the conditions tested. However, we recommend titrating the plasmid dosage in each experimental context, as optimal levels may vary depending on cell type, transfection efficiency, and other variables. The level of inhibition can be finely tuned by adjusting the amount of CHMP-NS3 plasmid. For an example of such optimization please refer to Wang et al.^1^

1. Day 1: Instructions for Cell Seeding.
  a. Seed 6∗10^5^ HEK-293T cells in 8 mL of medium per dish.
  b. Incubate the cells at 37°C with 5% CO_2_ for 24 h.
2. Day 2: Procedure for co-transfecting HEK293 cells with Gag and CHMP-NS3-Green plasmids in P100 dishes.
  a. Steps for including appropriate baseline, experimental, and positive controls during transfection:
    i. Baseline Control: Transfect cells with DNA encoding only Gag to establish a reference for normal VLP release.
    ii. Experimental Control: Co-transfect with DNA encoding both Gag and CHMP-mut-NS3-Green to assess the inhibitory effect of full-length CHMP-NS3-Green proteins on VLP release.
    iii. Positive Control: Co-transfect with DNA encoding Gag and GFP-VPS4-E228Q to confirm complete inhibition of VLP release.
  b. Instructions for preparing and applying DNA/jetPRIME transfection mix:
    Prepare the DNA mix using the pCDNA 3.1 vector to achieve a total of 8 μg.
    i. Gag plasmid: 0.5 μg.
    ii. CHMP2A-NS3-green or CHMP2A-mut-NS3-green: 2 μg.
    iii. CHMP3-NS3-green or CHMP3-mut-NS3-green: 4 μg
    iv. CHMP4B-NS3-green or CHMP4B-mut-NS3-green: 1 μg.
    v. GFP-VPS4 E228Q: 1 μg.
  c. Dilute the DNA mix in 800 μL of jetPRIME buffer, vortex for 10 s, and briefly spin down.
  d. Add 16 μL of jetPRIME reagent (using a DNA/reagent ratio of 1:2), vortex for 1 s, and spin down.
  e. Incubate at 20°C for 10 min.
  f. Slowly and evenly add the transfection mix to dishes where the cells have settled, swirling gently during the addition.
  g. Incubate the cells at 37°C with 5% CO_2_ for 24 h.
3. Day 3: Protocol for protease inhibitor (Glecaprevir) treatment of transfected cells.
  a. Pre-warm the cell culture medium to 37°C.
  b. Dilute 20 μL of 10 mM Glecaprevir stock solution in 8 mL of the pre-warmed medium.
    **CRITICAL:** Always use a fresh batch of Glecaprevir and avoid freeze-thaw cycles to maintain its biological activity.
  c. Wash away the existing cell medium.
  d. Add either the DMSO or Glecaprevir-containing medium to the cells, swirling gently to mix.
  e. Incubate the cells at 37°C with 5% CO_2_ for 4 h.
  f. Collect the supernatant for VLP purification and retain the cells for processing through cell lysis to obtain whole-cell protein extracts.

### Procedure for evaluating expression and inhibitory activity on HIV-1 VLP release of full-length CHMPs-NS3-Green proteins

**Timing: 3 days (steps 4 to 7)**

This section details the process for whole cell extract preparation and VLP purification, along with the quantification of VLP release inhibition via Western blot analysis.

4. Day 1: Procedure for preparing whole-cell extracts from transfected HEK293 cells.
  a. Washing: Wash the cells three times with ice-cold DPBS
  b. Lysis: Add 500 μL Lysis buffer and 5 μL nuclease to the cells.
  c. Incubate on ice for 20 min.
  d. Cell harvesting:
    i. Gently scrape adherent cells using a plastic cell lifter.
    ii. Transfer the cell suspension to a precooled micro-centrifuge tube at 4°C.
  e. Spin the tube at 16,000 rpm for 5 min at 4°C.
  f. Supernatant Collection:
    i. Carefully transfer the supernatant, which contains the soluble proteins, to a fresh tube kept on ice.
    ii. Discard the pellet containing organelles.
  g. Sample preparation:
    i. Take 30 μL of the supernatant.
    ii. Mix it with 10 μL of 4x loading buffer.
  h. Denaturation: Boil the mixture for 10 min at 95°C to denature the proteins.
  i. Storage: Store the prepared samples at −20°C for up to one month.
  j. Western Blot: Load 10 μL of the prepared sample into an SDS-PAGE gel and proceed with Western blot analysis to detect specific proteins.

**Pause point:** Whole cell extracts can be stored at −20°C for up to one month.

5. Day 1: Steps for purifying virus-like particles (VLPs) from cell culture supernatants.
  a. Collect the cell culture supernatant and filter it through a 0.45 μm filter to remove any cellular debris.
  b. Layer the filtered supernatant onto a 1 ml 20% sucrose solution and centrifuge at 30,000 rpm for 2 h at 4°C using a swinging-bucket rotor.
  c. Carefully remove and discard the entire supernatant, leaving the viral-like particles (VLPs) at the bottom of the tube.
  d. Re-suspend the VLP pellet in 30 μL of PBS to maintain their stability.
  e. Prepare the sample by taking 24 μL of VLPs and add 8 μL of 4x loading buffer for Western blot analysis.
  f. Boil the VLP sample for 5 min at 95°C to denature the proteins.
  g. Store the prepared VLP samples at −20°C for up to one month.
  h. Load 10 μL of the VLP sample into an SDS-PAGE gel and perform Western blot analysis to assess the VLP release inhibition.

**Pause point:** VLPs can be stored at −20°C for up to two weeks.

**Figure 1:**
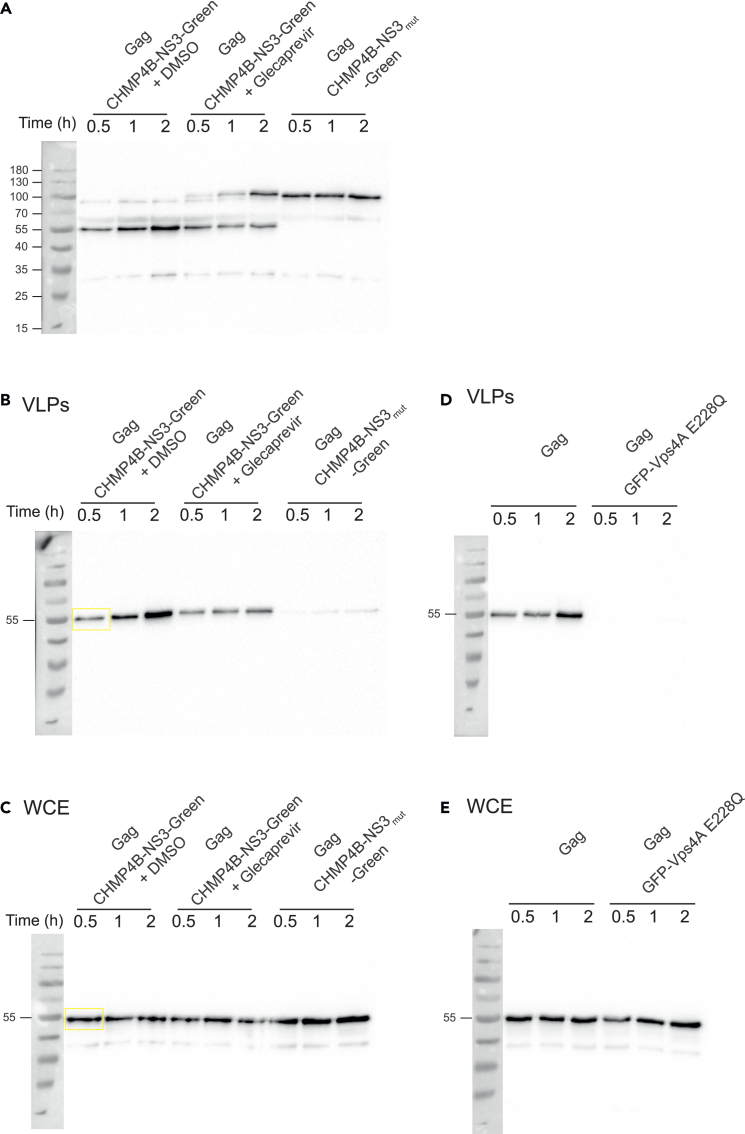
Inhibition of VLP release by CHMP4B-NS3-green—Immunoblot analysis (A–C) Inhibition of VLP release in cells co-transfected with Gag, CHMP4B-NS3-green (treated with either DMSO or Glecaprevir), and CHMP4B-NS3mut-green, as indicated. The time points correspond to the duration of drug treatment (Step 3f), with drug addition for 30 min, 1 h, and 2 h, respectively. (A) Immunoblot analysis showing the cellular expression of CHMP4B-NS3-green (treated with DMSO or Glecaprevir) and CHMP4B-NS3mut-green. (B) Immunoblot analysis of the HIV-1 VLP pellet. (C) Immunoblot analysis showing Gag HIV-1 cellular expression (WCE: whole cell extract). (B-C) A yellow square was added to highlight an example of the bands selected. (D and E) Inhibition of VLP release in cells co-transfected with either Gag and Vps4B E228Q or Gag alone, as indicated. (D) Immunoblot analysis of the HIV-1 VLP pellet. (E) Immunoblot analysis of Gag HIV-1 cellular expression (WCE: whole cell extract). The time points correspond to the duration of Step 3f. In each immunoblot, the first lane represents the molecular weight protein ladder. HIV-1 Gag proteins were detected using an anti-p24 antibody (B-E), while CHMP4B-NS3-green was detected using an anti-Flag antibody (A).

**Figure 2:**
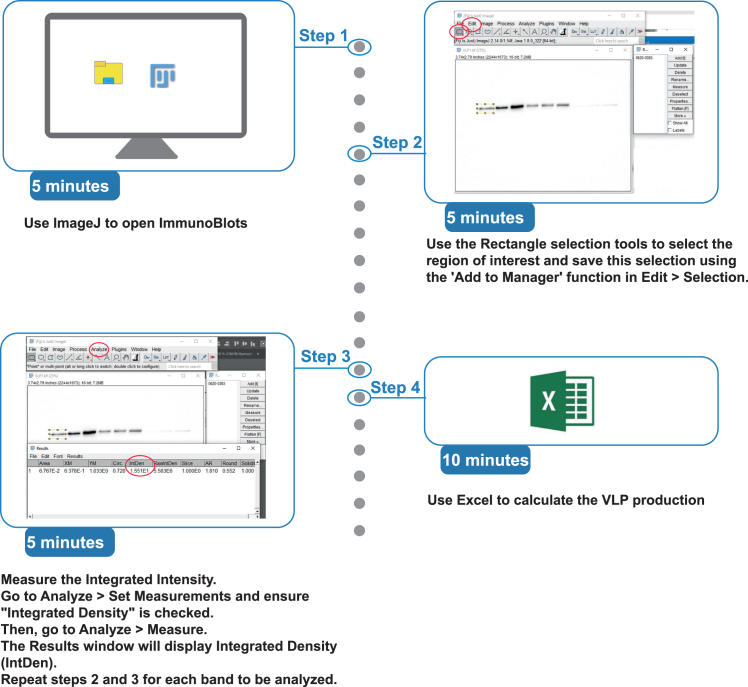
Workflow for immunoblot analysis using Fiji

6. Days 2-3: Protocol for performing SDS-PAGE and transferring proteins to a nitrocellulose membrane.
  a. Run a 10% Gel electrophoresis (SDS-PAGE) to separate proteins.
    i. Prepare a 10% SDS-PAGE gel:
          Prepare 10% SDS-PAGE resolving and stacking solutions.
          Pour 5 mL of 10% SDS-PAGE resolving gel solution into a gel casting apparatus.
          Allow it to polymerize for at least 30 min.
          Pour 1.5 mL of 5% SDS-Polyacrylamide stacking gel solution.
          Install gel combs and allow it to polymerize for 30 min.
    ii. Load 10 μL of whole cell extract or VLP sample (steps 4 and 5) into the wells of the gel, alongside a molecular weight marker (protein ladder) in one lane for reference.
    iii. Fill the electrophoresis tank with running buffer (Tris-Glycine-SDS).
    iv. Set the power supply to 80 V for stacking gel (first 15 min) and then 120 V for resolving gel until the dye front reaches the bottom (∼1 h).
  b. Instructions for Protein Transfer to Nitrocellulose Membrane.
    i. Assemble the transfer sandwich in the following order (from bottom to top) on a transfer cassette:
          Four sheets of Whatman 3MM paper (pre-wet with transfer buffer).
          Nitrocellulose membrane (pre-wet with transfer buffer).
          Gel.
          Four sheets of Whatman 3MM paper (pre-wet with transfer buffer).
    ii. Check for and remove any bubbles within the layers. Place the cassette into a transfer tank.
    iii. Perform semi-dry electro-transfer: 20 V for 20 min.
  c. Steps for blocking the membrane.
    i. Remove the membrane from the transfer system.
    ii. Wash it in PBST for 10 min.
    iii. Block the membrane with PBST milk for 1 h at 20°C on a rocking platform to prevent non-specific binding.
    iv. Wash the membrane five times with PBST for 5 min each.
  d. Procedure for performing Primary antibody incubation
    i. Prepare the primary antibody solution by diluting it 1:1000 in PBST buffer.
    ii. Incubate the membrane overnight at 4°C with gentle shaking to allow specific antibody binding.
    iii. Wash the membrane five times with PBST for 5 min each on a rocking platform to remove unbound antibodies.
  e. Procedure for performing Secondary antibody incubation
    i. Prepare the secondary antibody (HRP-conjugated) by diluting it 1:5000 in PBST buffer.
    ii. Incubate the membrane for 1 h at 20°C with gentle shaking.
    iii. Wash the membrane five times with PBST for 5 min each to remove excess secondary antibody.
  f. Guidance on detecting proteins using chemi-luminescence and capturing blot images.
    i. Prepare Enhanced Chemi-luminescence (ECL) reagent according to manufacturer’s instructions.
    ii. Incubate the membrane in ECL substrate for 1 min to allow signal development.
    iii. Use a chemiluminescent imaging system with an exposure time of 5 min.
    iv. Save the image in a TIF format for analysis.
7. Day 3: Steps for quantifying VLP release inhibition using band intensity analysis in Fiji (Figures 1 and 2).
  ***Note:*** Evaluate the expression of cleaved and uncleaved CHMP-NS3-Green proteins by performing a Western blot on whole-cell extracts, and detect the proteins using a primary anti-FLAG antibody (see Figure 1A for an example).
  ***Note:*** The transfected pCG-GagRevInd-7ires-puro plasmid encodes Gag but omits the HIV-1 protease gene.^36^ As a result, Western blot analysis detects only unprocessed Gag products (Figures 1B and 1C). Transfecting cells with CHMP-NS3-Green or Vps4B-E228Q does not cause Gag degradation compared to Gag-only controls, indicating that these constructs do not interfere with Gag stability or quantification (Figures 1B–1E).
  a. Open the Western blot image in Fiji (ImageJ 1.54f**^37^**) (Figure 2).
  b. Use the rectangular selection tool to define a consistent ROI for each Gag band (Figure 2).
  c. Measure the intensity of the Gag band in the VLP lane.
  d. Measure the intensity of the Gag band in the WCE lane.
  e. Calculate total Gag: (VLP intensity + WCE intensity).
  f. Compute VLP production using the formula:
    $$VLP\;production=\frac{Gag\;band\;value\;\left(in\;VLPs\right)}{Gag\;band\;value\;\left(in\;WCE\right)+Gag\;band\;value\;\left(in\;VLPs\right)}$$
  g. Normalize results by assigning a baseline value of 1 to control samples transfected with Gag alone.

### Steps for co-transfecting Gag and CHMP-NS3-Green plasmids into HeLA cells and applying protease inhibitor treatment in glass-bottomed micro dishes

**Timing: 2 days for step 8**

***Note:*** HeLa cells are well suited for studying HIV-1 VLP release through live-cell imaging, thanks to their rapid growth, efficient transfection, and flat morphology that improves imaging quality.

**CRITICAL:** Decontaminate the hood, bench surface, microscope stage and pipettes with 70% ethanol prior to use to prevent any potential contamination.

8. Days 1 and 2: Procedure for co-transfecting HeLa cells with Gag and CHMP-NS3-Green plasmids in glass-bottomed micro dishes.
  ***Note:*** Culture the cells in glass-bottom micro-dishes, specifically optimized for compatibility with advanced microscopy techniques (see key resources table for reference).
  a. Steps for coating glass-bottomed micro-dishes with poly-lysine to optimize live-cell imaging.
    i. Add 1 mL of poly-lysine solution to each culture micro-dish, ensuring the entire surface is covered.
    ii. Gently swirl the dish to distribute the solution evenly.
    iii. Incubate the culture dishes at 20°C for 4 h. Alternatively, you can incubate them at 4°C overnight for a stronger coating.
    iv. After incubation, remove the poly-lysine solution from the dishes.
    v. Rinse the dishes gently three times with sterile PBS to remove any unbound poly-lysine.
  b. Procedures for seeding HeLa cells for optimal transfection conditions.
    i. Seed 0.8∗10^5^ HeLa cells in 2 mL of medium per dish.
    ii. Incubate the cells at 37°C with 5% CO_2_ for 24 h.
  c. Steps for co-transfecting HeLa cells with Gag and CHMP-NS3-Green plasmids using jetPRIME.
    ***Note:*** To prevent morphological defects in VLPs caused by Gag-mCherry expression alone, untagged Gag was co-transfected.
    i. Guidance on including appropriate experimental and control conditions during transfection.
      Auto-fluorescence Control: Transfect cells with a DNA vector that does not encode any proteins (e.g., pCDNA3.1) to assess background fluorescence.
      Baseline Control for VLP Budding Localization: Transfect cells with DNA encoding only Gag and Gag-mCherry to establish a reference for normal VLP budding distribution.
      Experimental Control for Impaired CHMP Disassembly and Remodeling: Transfect cells with DNA encoding Gag, Gag-mCherry, and either CHMP-mut-NS3-Green or GFP-VPS4-E228Q to evaluate VLP budding localization under conditions that disrupt CHMP remodeling.
    ii. Instructions for preparing and applying DNA/jetPRIME transfection mix.
      Prepare the DNA mix using the pCDN 3.1 vector to achieve a total of 2 μg:
      Gag plasmid: 0.5 μg.
      Gag-mCherry: 0.1 μg.
      CHMP2A-NS3-green or CHMP2A-mut-NS3-green or CHMP2A-NS3-blue: 0.5 μg.
      CHMP3-NS3-green or CHMP2A-mut-NS3-green or CHMP3-NS3-blue: 0.5 μg.
      CHMP4B-NS3-green or CHMP2A-mut-NS3-green or CHMP4B-NS3-blue: 0.5 μg.
      GFP-VPS4 E228Q: 0.5 μg.
      Adjust the DNA mix with pCDNA 3.1 vector to achieve a final total of 2 μg.
    iii. Dilute the DNA mix in 200 μL jetPRIME buffer, vortex for 10 s and briefly spin down.
    iv. Add 4 μL jetPRIME reagent (using a DNA/reagent ratio of 1:2), vortex for 1 s, spin down.
    v. Incubate at 20°C for 10 min.
    vi. Slowly add the transfection mix to the cells, swirling gently during the addition.
    vii. Incubate the cells at 37°C with 5% CO_2_ for 24 h.
  d. Day 2: Instructions for preparing and applying protease inhibitor Glecaprevir to assess VLP budding inhibition.
    i. Pre-warm the cell culture medium to 37°C.
    ii. Dilute 5 μL of 10 mM Glecaprevir stock solution in 2 mL of the pre-warmed medium.
      **CRITICAL:** Always use a fresh batch of Glecaprevir and avoid freeze-thaw cycles to maintain its stability and effectiveness.
    iii. Prepare a negative control by adding 5 μL of DMSO to 2 mL of pre-warmed medium.
    iv. Wash away the existing cell medium.
    v. Add either the DMSO or Glecaprevir-containing medium to the cells, swirling gently to mix.
    vi. Incubate the cells at 37°C with 5% CO_2_ for 2 h.

### Steps for performing live-cell time-lapse TIRF microscopy to visualize HIV-1 VLP release at the plasma membrane

**Timing: 2 days for step 9**

9. Day 3: TIRF Microscopy Setup and Imaging.
  ***Note:*** We employed the optical sectioning capabilities of Total Internal Reflection Fluorescence Microscopy (TIR-FM) to minimize the strong diffuse fluorescence from cytoplasmic Gag-mCherry (see materials and equipment for detailed description of our experiment set up).
  **CRITICAL:** Pre-warm the microscope to 37°C overnight before imaging to minimize thermal fluctuations and ensure optimize operating conditions.
  a. Place the glass-bottom dish onto the microscope stage and set the CO_2_ level to 5%.
  b. Begin by using widefield fluorescence mode to locate cells co-transfected with green (CHMP-NS3-green or GFP-VSP4-E228Q) and red (Gag-mCherry) fluorescence on the glass surface.
  c. Switch to TIR-FM mode and adjust the focus carefully to bring the plasma membrane into sharp view.
  d. Fine-tune the TIR-FM angle to optimize the signal-to-noise ratio, ensuring that only the basal surface of the cells is illuminated.
  e. Adjust the laser intensity and exposure time to achieve an optimal balance between signal clarity and minimal photobleaching and phototoxicity.
  f. Define the region of interest (ROI) for imaging to reduce data size and focus on one or two cells of interest.
  g. Set the frame rate to one image every 450 ms over a 10-min duration.

### Guidance on analyzing HIV-1 VLP release kinetics using time-lapse video microscopy datasets

**Timing: 3 h for steps 10 to 12**

This section outlines the tracking of HIV-1 VLPs (labeled with Gag-mCherry) and provides the subsequent statistical workflow for analyzing the resulting dataset.

**Figure 3:**
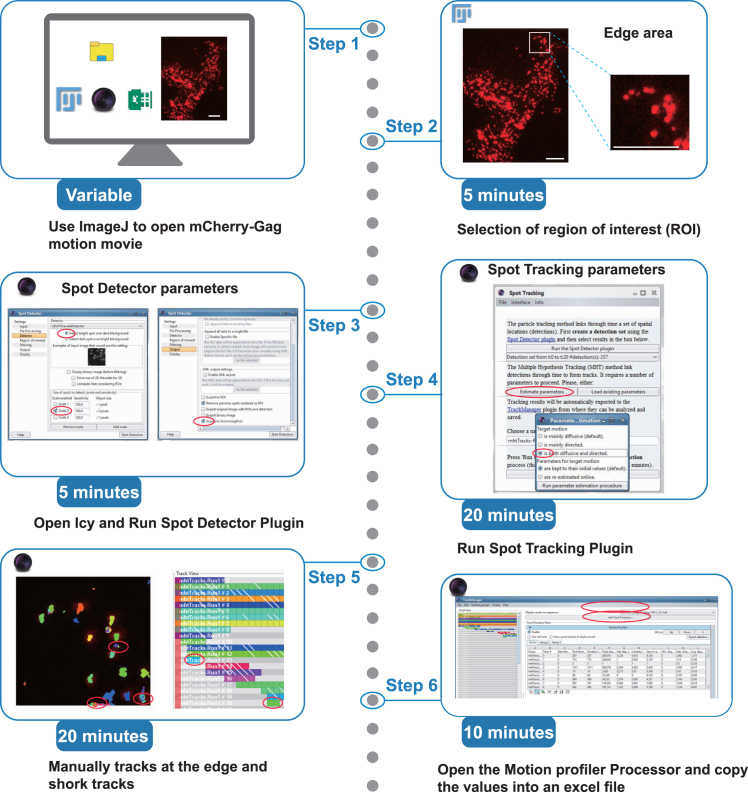
Workflow for VLP tracking. Scale bars represent 5 microns

**Figure 4:**
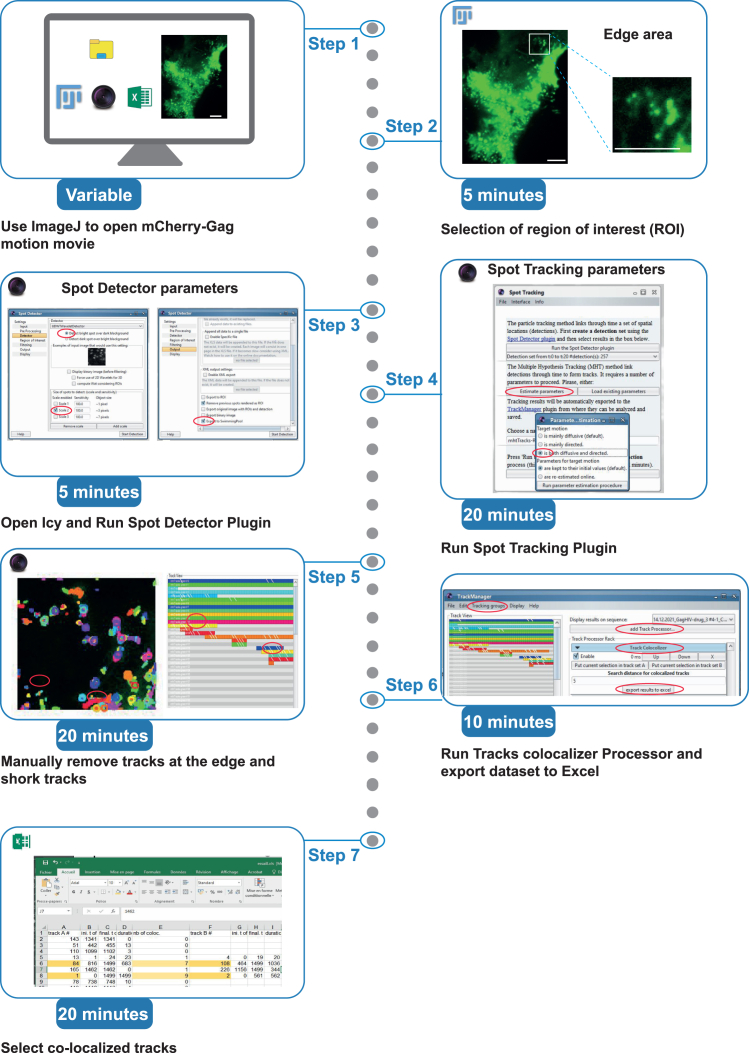
Workflow for co-localized Gag-mCherry and CHMPs-NS3-Green spot tracking Scale bars indicate 5 microns.

**Figure 5:**
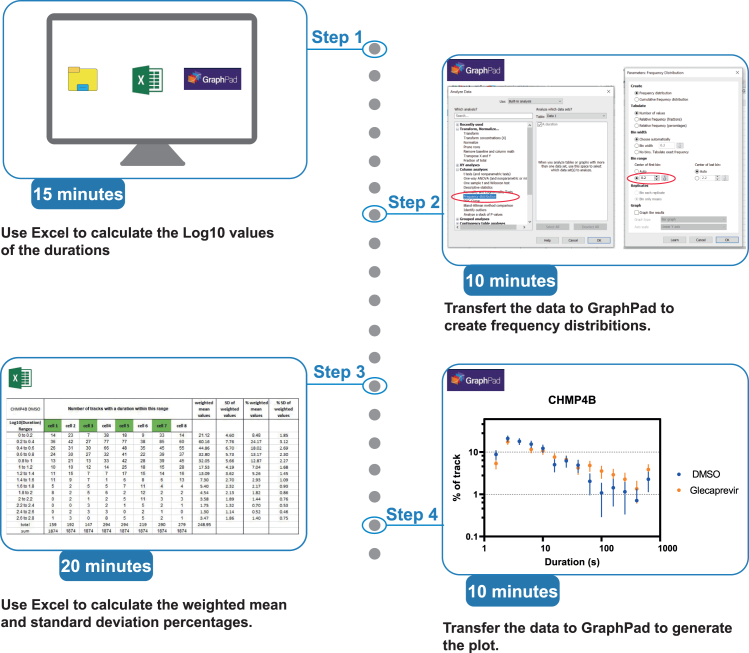
Workflow for statistical analysis Error bars represent standard deviation.

10. Steps for detecting and tracking HIV-1 VLPs labeled with Gag-mCherry using Fiji and Icy (Figure 3).
  a. Open the time-lapse movie in Fiji, focusing on the Gag-mCherry signal.
  b. Adjust the frame brightness and contrast as needed navigate to Image > Adjust > Brightness/Contrast.
  c. Improve image clarity by applying background subtraction: navigate to Process > Subtract Background > Sliding Paraboloid (Rolling Ball Radius: 50.0 pixels).
  d. Define the Region of Interest (ROI) in the -Gag-mCherry channel.
    **CRITICAL:** Select a region of interest (ROI at the edge of HeLa cells, where VLPs are clearly distinguishable from the background in each frame. Choose an ROI with minimal particle overcrowding (see also Troubleshooting 4).
  e. Save the cropped movie with the selected ROI applied.
  f. Save the ROI position by navigating to Edit > Selection > Add to Manager.
  g. In Icy, open the saved ROI movie for VLP tracking analysis.
  h. In Icy Run the Spot Tracking Plug-In:
    i. Run the Spot Detector. Use the parameters provided in Figure 3, ensuring that you enable the “export to the swimming pool” option.
    ii. In the Spot Tracking window, run the “Estimate Parameters” option. Select the target motion type as both diffusive and directed.
    iii. Execute the Icy Spot Tracking Plug-In. Upon completion, a Track Manager window will appear.
  i. Remove Problematic Tracks: Identify track shorter than three time frames, as well as those that detect particles at the edge of the ROI, and manually delete them from the track manager window Figure 3).
  j. In the track manager window, add the Track Processor plug-in called “Motion profiler”.
  k. Export Data: Copy the resulting values into an Excel file for further analysis.
    **CRITICAL:** This analysis should be performed under each condition for at least 8 cells from three independent culture experiments.
11. Steps for tracking CHMP-NS3-Green particles and analyzing their co-localization with Gag-mCherry-labeled VLPs (Figure 4).
  a. Open the time-lapse movie in Fiji, focusing on the CHMP-NS3-Green signal.
  b. Adjust the frame brightness and contrast as needed. Then, enhance improve image clarity by applying background subtraction: navigate to Process > Subtract Background > Sliding Paraboloid (Rolling Ball Radius: 50.0 pixels).
  c. Apply the ROI from the Gag-mCherry channel using the ROI Manager window.
  d. Save the cropped movie with the selected ROI applied.
  e. In Icy, open the saved ROI movie for VLP tracking analysis.
  f. In Icy Run the Spot Tracking Plug-In:
    i. Run the Spot Detector. Use the parameters provided in Figure 3, ensuring that you enable the “export to the swimming pool” option.
    ii. In the Spot Tracking window, run the “Estimate Parameters” option. Select the target motion type as both diffusive and directed.
    iii. Execute the Icy Spot Tracking Plug-In. Upon completion, a Track Manager window will appear.
  g. Remove Problematic Tracks: Identify track shorter than three time frames, as well as those that detect particles at the edge of the ROI, and manually delete them from the track manager window (Figure 4).
  h. In the track manager window.
    i. Add the Track Processor plug-in called “Track Colocalizer.” You may need to download this plug-in from the Icy website (https://icy.bioimageanalysis.org).
    ii. Select the Gag-mCherry tracking group, choose all tracks, and assign the current selection to Track Set A.
    iii. Select the CHMP-NS3-Green tracking group, choose all tracks, and assign the current selection to Track Set B.
    iv. Select both Gag-mCherry and CHMP-NS3-Green tracking groups.
    v. Export results to excel.
    vi. Wait for the export process to complete before opening the Excel file.
    vii. Open the Excel file and identify tracks with a co-localization count greater than five time frames.
12. Procedure for calculating and plotting VLP retention time distributions from tracking datasets (Figure 5).
  ***Note:*** Spot tracking generates a large dataset of thousands of tracks, with durations ranging from fractions of a second to up to 10 min. To better identify potential extensions in VLP retention at the plasma membrane, it is advisable to analyze the dataset using logarithmic scales on both the X and Y-axes.
  a. For each cell’s VLP track dataset, extract the tracking duration and convert it to seconds.
  b. For each track, calculate the Log10 value of its duration. This step is essential because GraphPad does not support direct use of XY logarithmic scales.
  c. Use GraphPad Prism software to create a frequency distribution of Log10 values of particle tracking durations with intervals of 0.2 (Figure 5).
  d. Calculate the weighted mean value for each interval and determine the standard deviation (SD) of these weighted means. Convert both the weighted means and their SDs into percentages.
  e. Plot these percentages on a logarithmic scale using GraphPad.

## Expected outcomes

The assay outlined in this protocol offers researchers a robust and precise tool for temporally controlling HIV-1 VLP release inhibition in various cell lines. This approach utilizes CHMP2A and CHMP4B proteins fused to the Hepatitis C NS3 protease, which behave like wild-type proteins due to auto-cleavage. In the presence of the protease inhibitor Glecaprevir, non-cleaved CHMP2A-NS3 and CHMP4B-NS3 inhibit budding by preventing membrane fission, resulting in delayed VLP release and subsequent accumulation. Conversely, an increased retention time at the plasma membrane served as a reliable indicator of budding inhibition.^1^^,^^39^

This assay not only ensures the ability to control the onset of CHMP-mediated inhibition of VLP release at designated time points post-transfection, but also allows for the initial expression of wild-type CHMP without disrupting ESCRT-III function or cell viability.^1^ Furthermore, by using either CHMP2A or CHMP4B-NS3 constructs, researchers can precisely control the timing of VLP inhibition at different entry points of the process.

The method leverages a transient transfection strategy, which has been successfully validated across a spectrum of both adherent and suspension cell types, including HEK293, HeLa CCL2, HeLa Kyoto, and FreeStyle cells.^1^

The versatility of this approach extends to the modulation of inhibition levels. By varying the amount of CHMP-NS3 DNA introduced during transfection, researchers can fine-tune the extent of inhibition, allowing for detailed exploration of the relationship between CHMP expression and ESCRT-III function during HIV-1 budding.^1^ Data gathered through this protocol demonstrate that the timing of un-cleaved CHMP2A, CHMP3 and CHMP4B protein expression begins as early as one hour following drug treatment, with peak expression times varying for different CHMP isoforms—24 h for CHMP4B and 4 h for CHMP2A and CHMP3.^1^ This temporal flexibility makes the assay an invaluable tool for studying the kinetics of ESCRT-III-driven processes in real time.

## Limitations

Researchers should consider several limitations. Glecaprevir treatment results in the accumulation of cleaved protein products, such as NS3-FP and CHMP proteins, which persist alongside full-length CHMP-NS3 proteins due to their prolonged lifespan. These cleavage products may interact with native CHMP, potentially complicating the interpretation of results. Thus, careful monitoring of cleaved versus uncleaved CHMP-NS3 protein levels is essential.^1^

Additionally, the irreversible nature of Glecaprevir-induced effects presents a further limitation. Upon treatment with Glecaprevir, full-length CHMP-NS3 proteins become resistant to NS3-mediated proteolysis and remain stable for up to 24 h.^1^ This prolonged stability prevents the use of this protocol in time-rescue experiments. Despite these constraints, the assay remains a powerful and adaptable tool for investigating the temporal dynamics of ESCRT-III activity.

## Troubleshooting

### Problem 1

Insufficient CHMP2A, CHMP3 or CHMP4B protein expression (visible after immunoblotting step 6).

### Potential solution

The condition of the effector cells, such as HEK293 cells, is also critical for successful transfection and robust CHMP-NS3 protein expression. Improving low expression levels may involve optimizing cell confluence prior to transfection, adjusting the amount of DNA transfected, or consistently using early-passage frozen stocks of HEK293 cells that are thawed monthly. Careful monitoring of the cells during the experiment is crucial for maintaining overall cell viability and maximizing protein expression.

One common issue is low expression of CHMP proteins. Endotoxins present in plasmid DNA preparations from Gram-negative bacteria can cause this effect, as these bacteria produce large amounts of lipopolysaccharides (LPS) in their membranes. These amphiphilic endotoxins can dramatically reduce both transfection efficiency and cell viability (Step 2 and 8). To mitigate this, we recommend using endotoxin-free plasmid DNA preparation methods or commercially available kits designed to remove endotoxins during DNA isolation (see key resources table for reference).

Additionally, consider generating a stable cell line expressing CHMP constructs, as they do not compromise cell viability.^1^ This approach minimizes cell-to-cell variability, a common issue in transient transfection experiments, and ensures a more consistent, reproducible expression system for long-term studies.

### Problem 2

Low VLP release inhibition (visible in step 6 and 7).

### Potential solution

Generating a stable cell line expressing CHMP constructs will minimize cell-to-cell variability and reduce inconsistencies caused by transfection efficiency.

Glecaprevir, a temperature-sensitive compound, plays a vital role in this assay (Step 3 and 8). Proper handling is essential to prevent degradation and maintain its efficacy. For optimal results, dissolve Glecaprevir in DMSO and store aliquots at −20°C for up to one month or at −80°C for up to six months. To avoid loss of potency, refrain from repeated freeze-thaw cycles. When stored as a powder, Glecaprevir remains stable for up to three years at −20°C and for two years at 4°C. Following these guidelines ensures effective inhibition of VLP release during the assay.

### Problem 3

Variations in VLP release due to cell line and genetic background (visible in step 6 and 7).

Some human cell lines, such as HeLa cells (step 8), express Bst2/tetherin, a transmembrane protein with a GPI anchor that physically tethers various enveloped viruses, including HIV-1, to the surface of infected cells.^40^^,^^41^^,^^42^^,^^43^ This can result in undetectable HIV-1 virion release by immunoblotting.^40^

### Potential solution

To circumvent this issue, we recommend using cell lines that do not express Bst2 (also known as tetherin and CD317), such as HEK293, HT1080, or Cos7 cells.^40^ Another effective approach is to co-transfect with the HIV-1 accessory protein Vpu (see key resources table), which effectively downregulates Bst2 from the cell surface, facilitating virion release.^4^^,^^40^^,^^41^^,^^44^^,^^45^^,^^46^ A third approach involves using Bst2 knockout cells, such as those described in,^1^^,^^47^ where Bst2 protein expression has been abolished.

### Problem 4

Variations in Spot tracking due to differences in region of interest selection (related to Step 10).

### Potential solution

To ensure reliable and accurate outcomes for your particle tracking experiment, consider implementing the following potential strategies.

• Select a region of interest (ROI) where VLPs are clearly distinguishable from the background in each frame.
• Position the ROI at the edge of HeLa cells, where the plasma membrane is thinnest and exhibits minimal movement, ensuring accurate particle detection over time.
• Ensure reliable linkage of detected particles across frames. To enhance tracking accuracy, choose an ROI with minimal particle overcrowding, reducing tracking errors and improving linkage accuracy throughout the video sequence.

## Resource availability

### Lead contact

Further information and requests for resources and reagents should be directed to Cécile Boscheron (cecile.boscheron@ibs.fr).

### Technical contact

Technical questions on executing this protocol should be directed to Cécile Boscheron (cecile.boscheron@ibs.fr).

### Materials availability

Plasmids generated in this study have been deposited to Addgene (see key resources table for reference).

### Data and code availability

• Spot tracking datasets are accessible at https://doi.org/10.57745/69UNAM.
• This study did not generate code.

## Acknowledgments

We thank Arnaud Echard for the generous gift of cells. H.W. acknowledges scholarship support from the China Scholarship Council (CSC). W.W. acknowledges support from the Institut Universitaire de France (IUF). C.B. acknowledges support from ANRS (ANRS0654). C.B. and H.W. acknowledge support from GRAL (CF 7C047GRAL). C.B. and W.W. acknowledge access to the platforms of the Grenoble Instruct-ERIC center (IBS and ISBG; UAR 3518 CNRS-CEA-UGA-EMBL) within the Grenoble Partnership for Structural Biology (PSB), with support from FRISBI (ANR-10-INBS-05-02) and GRAL, a project of the University Grenoble Alpes graduate school (Ecoles Universitaires de Recherche) CBH-EUR-GS (ANR-17-EURE-0003). We thank the HIV Reagent Program, Division of AIDS, NIAID, NIH, for providing pcDNA-Vphu, Anti-Bst2, and Anti-HIV-1 p24. We thank Jean-Philippe Kleman for support at the IBS M4D microscopy platform. IBS acknowledges integration into the Interdisciplinary Research Institute of Grenoble (IRIG, CEA). We thank the Microcell core facility from the Institute for Advanced Biosciences (UGA - Inserm U1209 - CNRS 5309). This facility is part of the Imagerie-Sciences du Vivant – In vitro labeled by the GIS-IBISA and is member of the national infrastructure France-BioImaging supported by the French National Research Agency (ANR-10-INBS-04).

## Author contributions

Conceptualization, W.W.; investigation, H.W. and C.B.; writing – original draft, C.B.; writing – review and editing, W.W., H.W., and C.B.; funding acquisition, W.W. and C.B.; supervision, W.W. and C.B.

## Declaration of interests

The authors declare no competing interests.

## Declaration of generative AI and AI-assisted technologies in the writing process

During the preparation of this work, the author used ChatGPT in order to improve syntax and verify spelling accuracy. After using this tool, the authors reviewed and edited the content as needed and take full responsibility for the content of the publication.
